# Generation of an Oncolytic Herpes Simplex Viral Vector Completely Retargeted to the GDNF Receptor GFRα1 for Specific Infection of Breast Cancer Cells

**DOI:** 10.3390/ijms21228815

**Published:** 2020-11-21

**Authors:** Bonnie L. Hall, Daniela Leronni, Yoshitaka Miyagawa, William F. Goins, Joseph C. Glorioso, Justus B. Cohen

**Affiliations:** 1 Department of Microbiology and Molecular Genetics, University of Pittsburgh, Pittsburgh, PA 15219, USA; DAL118@pitt.edu (D.L.); yoshitaka-miyagawa@nms.ac.jp (Y.M.); goins@pitt.edu (W.F.G.); glorioso@pitt.edu (J.C.G.); 2 Department of Neurological Surgery, University of Pittsburgh, Pittsburgh, PA 15219, USA; 3 Department of Biochemistry and Molecular Biology, Nippon Medical School, Tokyo 113-0031, Japan

**Keywords:** oncolytic, herpes simplex virus, breast cancer

## Abstract

Oncolytic herpes simplex viruses (oHSV) are under development for the treatment of a variety of human cancers, including breast cancer, a leading cause of cancer mortality among women worldwide. Here we report the design of a fully retargeted oHSV for preferential infection of breast cancer cells through virus recognition of GFRα1, the cellular receptor for glial cell-derived neurotrophic factor (GDNF). GFRα1 displays a limited expression profile in normal adult tissue, but is upregulated in a subset of breast cancers. We generated a recombinant HSV expressing a completely retargeted glycoprotein D (gD), the viral attachment/entry protein, that incorporates pre-pro-GDNF in place of the signal peptide and HVEM binding domain of gD and contains a deletion of amino acid 38 to eliminate nectin-1 binding. We show that GFRα1 is necessary and sufficient for infection by the purified recombinant virus. Moreover, this virus enters and spreads in GFRα1-positive breast cancer cells in vitro and caused tumor regression upon intratumoral injection in vivo. Given the heterogeneity observed between and within individual breast cancers at the molecular level, these results expand our ability to deliver oHSV to specific tumors and suggest opportunities to enhance drug or viral treatments aimed at other receptors.

## 1. Introduction

Developing novel therapeutic approaches for the treatment of breast cancer is essential; in 2018, over 600,000 women worldwide died of breast cancer and over 2 million new breast cancer cases were reported [1]. Oncolytic viruses (OV) have been created from a variety of virus species and have been tested in human clinical trials for a broad array of solid tumors, including breast cancer [2,3,4,5,6]. The oncolytic HSV(oHSV) vector Imlygic received U.S. Food and Drug Administration approval in 2015 for treatment of melanoma [7] and clinical trial data demonstrated that Imlygic in conjunction with anti-checkpoint antibodies was even more effective than either therapy alone [8]. Our current understanding of the tumor microenvironment (TME) in both human breast cancer and immune-competent animal models also suggests that immune-modulatory therapeutics are key to OV success [9,10,11,12]. The large payload capacity of HSV makes it an excellent candidate for delivery of transgenes to augment the therapeutic potential of the virus [12,13].

Considerable effort has gone into devising strategies to achieve safety and efficacy for oHSV. Vector delivery has largely relied on intratumoral inoculation with off-target replication minimized by engineered mutations that reduce virulence [14]. An attractive alternative approach is vector retargeting, whereby virus infection is restricted to cells expressing tumor-associated cell surface receptors [15]. By limiting the potential for off-target cell transduction, this approach reduces the need for attenuating mutations. HSV vectors have been completely retargeted to a number of cell surface receptors, including epidermal growth factor receptor (EGFR/EGFRvIII) [16], epithelial cell adhesion molecule (EpCAM) [17], human epidermal growth factor receptor 2 (HER2) [18], and prostate specific membrane antigen (PSMA) [15]. We and others have shown that retargeted viruses can effectively treat tumors in multiple mouse model systems [16,19,20].

The step-wise process of HSV receptor binding and cell entry requires four essential envelope glycoproteins, gD, gB, and the heterodimer gH/gL. Virion gD binds to one of its cellular receptors (nectin-1, herpesvirus entry mediator (HVEM), or 3-*O*-sulfotransferase modified heparan sulfate), which activates gD through conformational changes that allow it to interact with and activate the gH/gL heterodimer, which in turn activates the fusogenic state of gB [21]. gB then mediates fusion of the viral envelope with the cell surface or endosomal membrane, allowing subsequent entry of the viral capsid into the host cell and trafficking to the nucleus. Retargeting of virus infection involves elimination of the interactions between gD and its cognate receptors, for example by mutation of gD residue 38 to ablate nectin-1 binding and replacement of N-terminal residues comprising the HVEM ligand with a ligand for a tumor-associated target receptor [17,22]. In the present study, we selected the glial cell line-derived neurotrophic factor (GDNF) receptor alpha 1 (GFRα1) as a target for retargeted oHSV infection. GFRα1 is a GPI-anchored cell-surface receptor that recognizes both GDNF and neurturin (NTN). Others have previously replaced the amino terminus of HSV-1 glycoprotein C (gC) with pre-pro-(pp)GDNF, removing the signal peptide (SP) and heparan sulfate-binding domain of gC and providing the SP and processing sites of ppGDNF, to favor virus attachment to GFRα1-expressing neurons [23,24]. While these studies demonstrated the functionality of ppGDNF as an HSV glycoprotein retargeting ligand, gC is dispensable for HSV entry, and, unlike gD retargeting, gC retargeting does not alter virus entry specificity.

GFRα1 is expressed at a low or undetectable level in most adult tissues but is expressed highly in breast tumor tissue [25]. In particular, GFRα1 expression was upregulated compared to normal breast tissue in almost 60% of breast cancer patients sampled and this upregulation was correlated with estrogen receptor (ER)-positive breast cancer cases [26,27]. ER^+^ breast cancer cases represent over 75% of all breast cancers and these patients have a long-term risk of death from the disease [28,29]. Breast cancer is a highly heterogenous group of cancers, exhibiting heterogeneity in morphology and at the molecular level. Breast cancer profiling of gene expression signatures has defined at least four groups of cancer patients with distinct HER2 and hormone receptor (HR: ER and/or progesterone receptor) expression levels: (1) luminal A: HR^+^/HER2^-^, (2) luminal B: HR^+^/HER2^+/-^, (3) HER2-overexpressing: HR^-^/HER2^+^, and (4) basal-like: HR^-^/HER2^-^, generally corresponding to triple negative [30]. Breast cancer-derived cell lines provide models of breast cancer that approximate features of the original tumor subtypes, being similarly grouped by receptor expression as: (1) luminal, (2) HER2^+^, or (3) triple negative [31]. Here, we demonstrate that GFRα1 is expressed in two luminal cancer cell lines (MCF7, HCC1500) and not in one HER2^+^ (MDA-MB-453) and one triple negative (MDA-MB-231) cell line.

Breast cancer treatment with completely retargeted oHSV provides a unique therapeutic approach to deliver robust oHSV vectors to the tumor with limited off-target virus infection that can likely be further restricted by transcriptional and translational control measures [32,33]. While highly promising HER2-retargeted oHSV vectors have been developed [15,18], the molecular heterogeneity observed between and within breast cancer cases underlines the need for a diversity of targeted treatments. In this report we demonstrate that HSV vectors can be completely retargeted for infection via GFRα1. The GFRα1-targeted vector efficiently transduced and killed human breast cancer cells bearing the receptor, while cells not expressing the receptor remained uninfected, even at high multiplicity of infection (MOI). Moreover, the retargeted vector induced tumor regression in an MCF7 subcutaneous flank tumor model in nude mice.

## 2. Results

### 2.1. Vector Engineering

**Figure 1:**
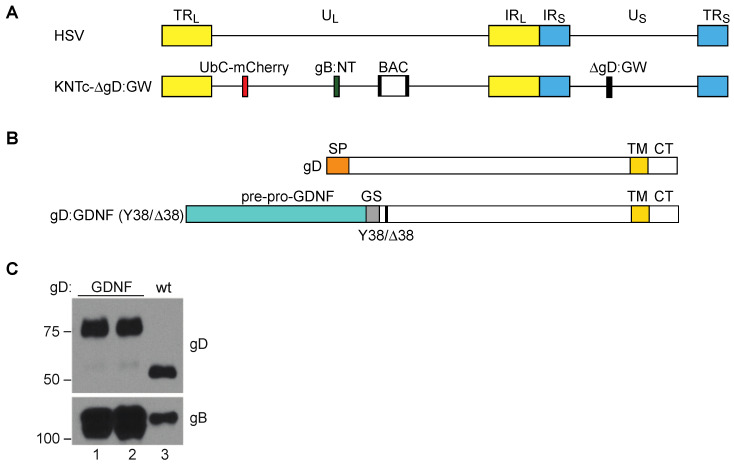
Vector engineering. (**A**) Schematic depiction of the herpes simplex virus (HSV) genome. Terminal repeats (TR_L_, TR_S_) of the unique long (U_L_) and unique short (U_S_) segment, respectively; inverted internal repeats (IR_L_, IR_S_) of the unique long (U_L_) and unique short (U_S_) segment, respectively. The KNTc-ΔgD:GW genome represented underneath contains loxP-flanked bacterial artificial chromosome (BAC) sequences, a ubiquitin C (UbC)-promoter driven mCherry cassette (UbC-mCherry), a GW cassette in place of the glycoprotein D (gD) coding sequence (ΔgD:GW), and mutations in glycoprotein B that enhance retargeted virus entry (gB:NT) [36]. (**B**) Illustration of wild type gD, depicting the approximate size and location of the signal peptide (SP), transmembrane domain (TM), and cytoplasmic tail (CT), and the recombinant gD proteins gD:GDNF and gD:GDNFΔ38; Y38/Δ38 refers to the wild type (wt) gD residue 38 (Y) or the deleted (∆) residue 38. In both recombinant proteins the pre-pro-GDNF ligand was tethered to the C-terminus of gD at amino acid 25 by a flexible glycine-serine spacer (GS; (G_4_S) × 3). (**C**) Western blot analysis of gD:wt and gD:GDNF proteins incorporated into purified virus particles. 1 × 10^8^ genome copies (gc) of purified virus particles were loaded per lane and blotted with either anti-gD or anti-gB antibodies. Lanes 1 and 2 represent virus stocks from two independent KNTc-gD:GDNF BAC isolates. The virus stock from lane 1 was used for the experiments pictured in Figure 2; the two isolates yielded comparable results.

All viral backbones generated for this study were based on the HSV-1 genome, strain KOS (Figure 1A), modified to contain loxP-flanked bacterial artificial chromosome (BAC) sequences for genome manipulation in bacteria [34]. The KNTc-∆gD:GW backbone [35] (Figure 1A), in addition contained an mCherry reporter gene controlled by the ubiquitin C (UbC) promoter between U_L_3 and U_L_4 for visualization of infected cells, two mutations in the gB gene previously shown to enhance retargeted virus entry [16,36], and a Gateway (GW) cassette in place of the gD coding sequence for orientation-specific introduction of all tested gD coding sequences.

To target GFRα1 for virus entry, we genetically replaced the signal peptide and HVEM binding N-terminal domain of gD with ppGDNF to create gD:GDNF, which maintained gD residue Y38 to preserve interaction with the HSV receptor nectin-1 (Figure 1B). Analogous to the previously described GFRα1-targeted gC [23,24], this design provided the ppGDNF SP and processing sites for N-terminal membrane translocation and exposure of mature GDNF, along with the downstream transmembrane domain (TM) of gD to anchor the protein in the viral envelope or cell membrane. We introduced the gD:GDNF coding sequence into the KNTc-∆gD:GW genome via LR clonase reaction in vitro and produced infectious KNTc-gD:GDNF virus by transfection of purified BAC DNA into nectin-1-expressing cells. Western blot analysis revealed the presence of gD:GDNF in purified virus particles as a higher molecular weight protein compared to wild-type (wt) gD (Figure 1C).

### 2.2. The GDNF Ligand Allows GFRα1-Specific Entry

**Figure 2:**
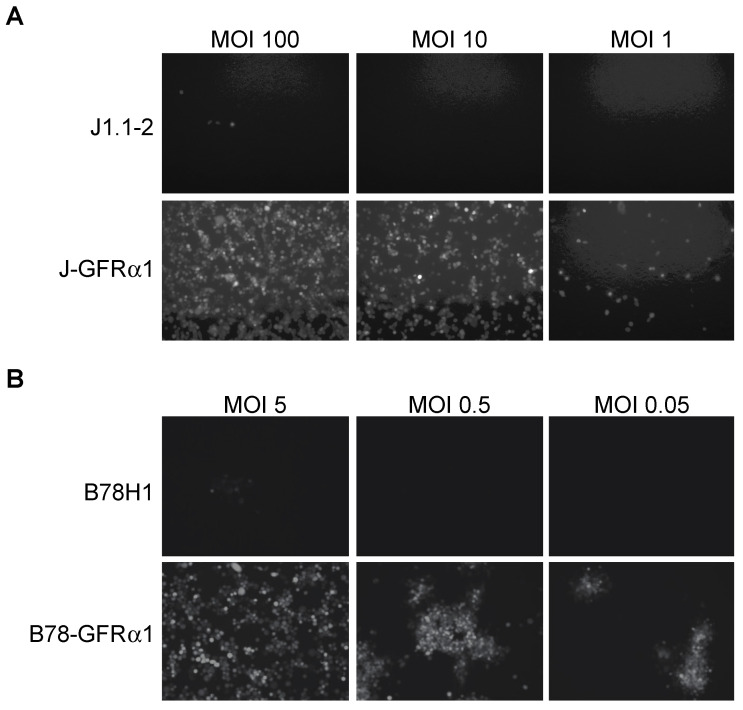
Receptor dependence of KNTc-gD:GDNF virus entry. Infection of (**A**) J1.1–2 and J-GFRα1 cells or (**B**) B78H1 and B78-GFRα1 cells with KNTc-gD:GDNF virus at the indicated multiplicities of infection (MOIs) based on pfu (plaque-forming unit) titers on GFRα1-transduced U2OS cells (U2OS-ICP4-GFRα1, Figure S1) (Table 1). Virus entry into cells was visualized at 24 h post infection (hpi) as mCherry fluorescence.

To assess the ability of purified KNTc-gD:GDNF virus to enter cells solely via interaction with GFRα1, we derived GFRα1-expressing cell lines from gD receptor (HVEM, nectin-1)-deficient J1.1-2 and B78H1 cells (Figure S1). After infection of J-GFRα1 cells at different MOIs, we recorded HSV-mediated mCherry fluorescence at 24 h post infection (hpi). KNTc-gD:GDNF virus was able to enter J-GFRα1 cells but did not enter parental J1.1-2 cells (Figure 2A). The number of virus-infected cells decreased in a MOI-dependent manner. Likewise, we observed viral reporter gene expression in GFRα1-transduced B78H1 cells, but not in the parental B78H1 cells (Figure 2B). These data demonstrated GFRα1-dependent, nectin-1-independent KNTc-gD:GDNF virus entry.

### 2.3. Fully Retargeted Virus Demonstrates GFRα1-Specific Entry

**Figure 3:**
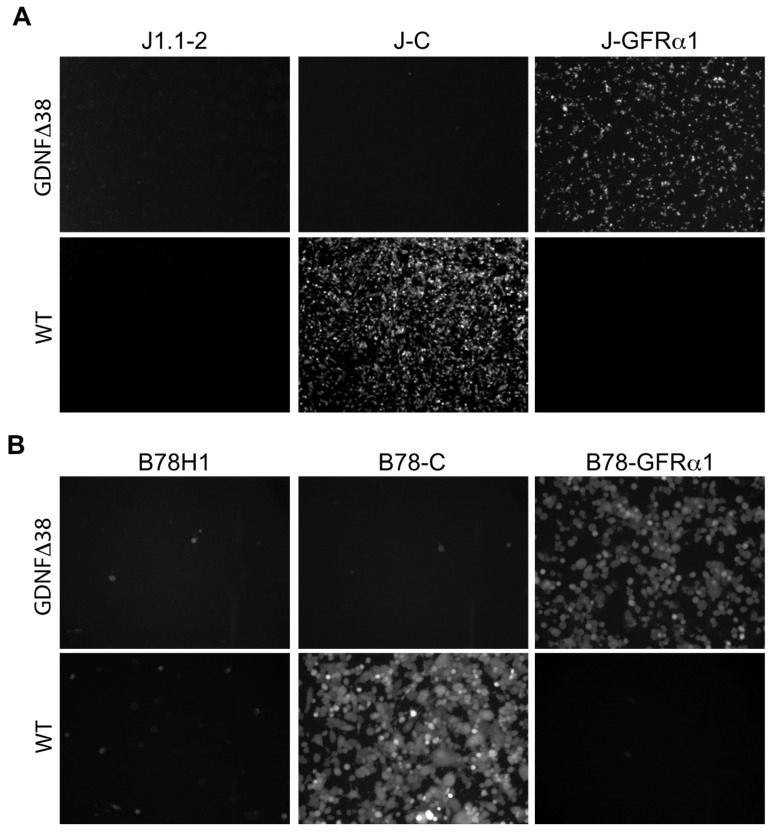
Receptor specificity of KNTc-gD:GDNFΔ38 virus entry. Infection of (**A**) J1.1-2, J–C (nectin-1^+^) and J-GFRα1 cells or (**B**) B78H1, B78-C and B78-GFRα1 cells with KNTc-gD:GDNFΔ38 and KNTc-gD:wt viruses at 0.5 pfu/cell. Virus entry into cells was visualized as mCherry fluorescence at 24 hpi.

To generate a nectin-1-detargeted version of the virus, we recombined the gD:GDNFΔ38 coding sequence, containing a deletion of gD residue 38 (Figure 1B), into KNTc-∆gD:GW BAC DNA and produced the corresponding virus, KNTc-gD:GDNFΔ38, by transfection of U2OS-ICP4-GFRα1 cells (Figure S1). The fully retargeted virus was able to replicate and spread in these cells and high-titer stocks were obtained (Table 1). We compared the receptor specificities of KNTc-gD:GDNFΔ38 and KNTc-gD:wt by infection of J1.1-2 and B78H1 cells transduced with the appropriate receptors (Figure 3). At 24 hpi, KNTc-gD:GDNFΔ38 showed entry into J-GFRα1 cells but not into nectin-1-transduced J1.1-2 (J–C) cells, while KNTc-gD:wt virus was able to enter J–C, but not J-GFRα1 cells (Figure 3A). Similar data were obtained from the comparable panel of B78H1 cells (Figure 3B). These results confirmed that the fully retargeted virus was no longer able to enter cells via the natural gD receptor nectin-1.

### 2.4. GFRα1-Retargeted Virus Infects and Kills Breast Cancer Cells in a GFRα1-Dependent Manner

**Figure 4:**
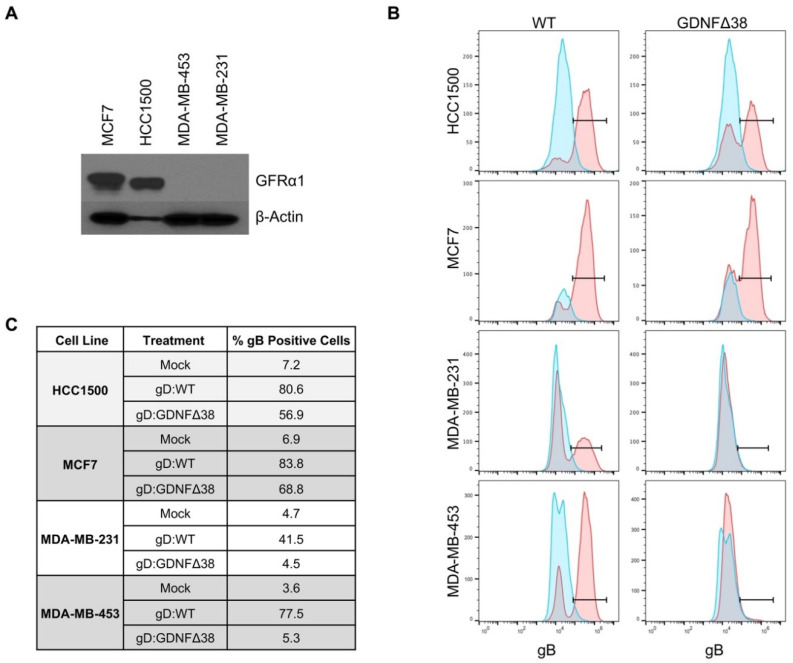
Virus infection of breast cancer cell lines. (**A**) MCF7, HCC1500, MDA-MB-231, and MDA-MB-453 cell lines were assessed for GFRα1 expression by Western blot analysis of whole cell lysates; β-actin detection was used as a loading control. (**B**) HCC1500, MCF7, MDA-MB-231, and MDA-MB-453 cells were infected with KNTc-gD:GDNFΔ38 or KNTc-gD:wt virus at 3 pfu/cell and flow cytometry for gB protein on the cell surface was performed at 24 hpi. For each cell line, uninfected cells are shown in blue compared to virus infected cells in red; KNTc-gD:wt infected cells (WT) and KNTc-gD:GDNFΔ38 infected cells (GDNFΔ38). (**C**) Quantification of gB^+^ cell populations as a percent of the total analyzed cells for each treatment group.

We chose four human breast cancer lines, MCF7, HCC1500, MDA-MB-453, and MDA-MB-231, and assessed GFRα1 expression by Western blot. HCC1500 and MCF7, both ER^+^ [31], expressed detectable levels of GFRα1 protein, while MDA-MB-231 and MDA-MB453, both ER^-^ [31], did not (Figure 4A), consistent with the reported association of ER and GFRα1 expression in human cancer patients [27]. We infected all four cell lines with KNTc-gD:GDNFΔ38 or KNTc-gD:wt virus and assessed infections by flow cytometry for gB, a late (γ1) viral gene product [37] that is incorporated into the infected cell membrane during viral replication (Figure 4B), and by mCherry visualization of infected cells (Figure S2). These data demonstrated that HCC1500 and MCF7 cells were permissive for both gD:wt and retargeted gD virus entry. Approximately 80% and 84% of HCC1500 and MCF7 cells, respectively, were infected by gD:wt virus, and 57% and 69% were infected by the retargeted virus (Figure 4C). In contrast, while MDA-MB-231 and MDA-MB-453 were infected by gD:wt virus (42% and 78%, respectively), infection by retargeted virus was minimal at best (≤5%, compare to mock).

**Figure 5:**
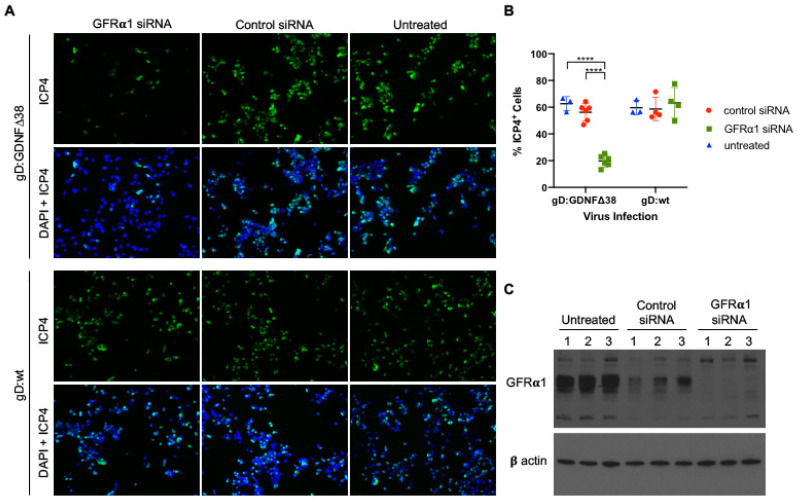
Virus infection of GFRα1 siRNA transduced cells. (**A**) MCF7 cells transfected with GFRα1-specific or non-specific (“Control”) siRNA pools were infected after 72 h with KNTc-gD:GDNFΔ38 (2 pfu/cell) or KNTc-gD:wt (0.2 pfu/cell) and stained at 6 hpi for ICP4 (upper rows) and DAPI (DAPI + ICP4, lower rows); representative images from triplicate infections are shown. (**B**) Quantification of the ICP4^+^ cell populations as a percentage of the total analyzed cells (DAPI) was performed for each treatment group using ImageJ software. Averages represent counts from 3–6 images ± SD; statistical differences were determined by two-way ANOVA (**** *p* < 0.0001). (**C**) Untreated and siRNA-treated MCF7 cells were assessed for GFRα1 protein expression by Western blot analysis of whole cell lysates; three biological replicates are shown for each condition. β-actin detection was used as loading control.

To strengthen the suggestion that GFRα1 expression is central to efficient retargeted virus infection of breast cancer cells, we transfected MCF7 cells with GFRα1-specific or non-targeting control short interfering RNA (siRNA) pools, exposed these and uninfected cells at 72 h to KNTc-gD:GDNFΔ38 (2 pfu/cell) or KNTc-gD:wt virus (0.2 pfu/cell), and assessed virus entry by staining for the viral immediate-early protein ICP4 at 6 hpi. As indicated by the representative images of Figure 5A and confirmed by the quantitative data of Figure 5B, the specific siRNA pool reduced entry of the gD:GDNFΔ38 virus, but not the gD:wt virus, while the control siRNA pool had no pronounced effect on entry by either virus. Western blot analysis at the time of virus infection (Figure 5C) confirmed that the specific siRNA pool dramatically reduced GFRα1 protein expression compared to untreated cells. The control siRNA pool also reduced GFRα1 protein levels, although to a lesser extent, but the data indicate that this reduction did not significantly affect entry by either virus. While these results do not fully exclude contributions from other receptors, they are in line with the conclusion that GFRα1 expression is the key to efficient infection of breast cancer cells by KNTc-gD:GDNFΔ38.

**Figure 6:**
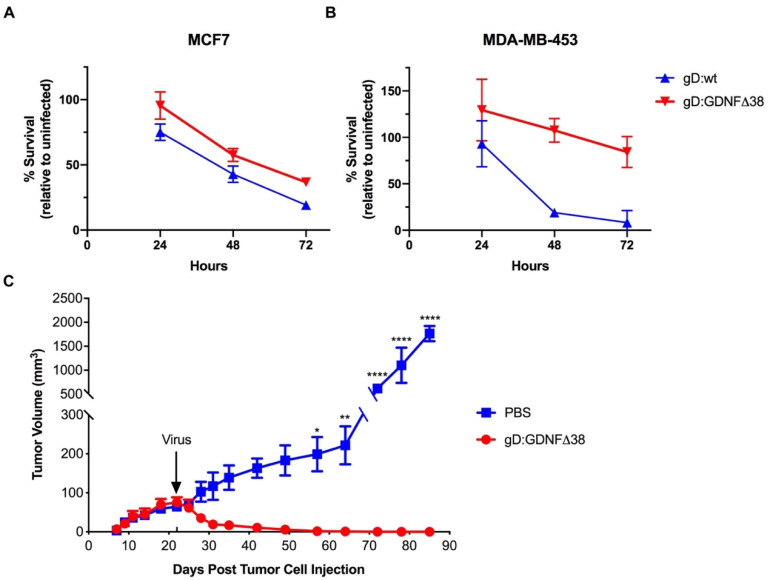
Virus-mediated cell death in vitro and tumor treatment. (**A**) MCF7 or (**B**) MDA-MB-453 cells were infected with KNTc-gD:GDNFΔ38 or KNTc-gD:wt virus at 3 pfu/cell and cell viability at 24, 48 and 72 hpi was measured by alamarBlue assay. Data are presented as the percentage of viable cells relative to uninfected cells at each time point. Averages presented at each time point represent 5–8 independent infections ± SEM. Statistics were determined by two-way ANOVA comparing virus infected cells to uninfected control cells at each time point. At 48 and 72 hpi, the viability of MCF7 cells infected with KNTc-gD:wt and KNTc-gD:GDNFΔ38 was significantly reduced compared to uninfected cells (KNTc-gD:wt, *p* < 0.0001 at 48 and 72 hpi, and KNTc-gD:GDNFΔ38, *p* = 0.0003 at 48 hpi and *p* < 0.0001 at 72 hpi). At 72 hpi, the viability of MDA-MB-453 cells infected with KNTc-gD:wt was significantly reduced compared to uninfected cells (*p* < 0.0001). The viability of MDA-MB-453 cells infected with KNTc-gD:GDNFΔ38 was not significantly different from that of uninfected cells at any time point tested. (**C**) MCF7 cells were implanted in the right hind flank in BALB/c athymic nude mice and tumors were injected with 1 × 10^8^ pfu of KNTc-gD:GDNFΔ38 or phosphate-buffered saline (PBS) when reaching a volume of approximately 70 mm^3^ (arrow, d22). Average tumor volumes in mm^3^ (mean ± SD of 3 animals/group) are presented over time. Statistical differences were determined by two-way ANOVA. KNTc-gD:GDNFΔ38 treated tumors were significantly reduced in volume compared to PBS-injected controls (d57, * *p* < 0.05; d64, ** *p* < 0.01; d72-d85, **** *p* < 0.0001).

We next assessed virus-mediated killing of MCF7 and MDA-MB-453 cells using the alamarBlue cell viability assay. The results of a 72-h time course demonstrated that both gD:wt and retargeted virus were cytotoxic for GFRα1-positive MCF7 cells, resulting in a significant reduction in cell viability over the 72-h time course (Figure 6A). In contrast, only the gD:wt virus was cytotoxic for GFRα1-negative MDA-MB-453 cells (Figure 6B). These data were consistent with primary dependence of KNTc-gD:GDNFΔ38 infection on host-cell GFRα1 expression.

### 2.5. GFRα1-Retargeted Virus Induces Tumor Regression in a Nude Mouse Model

We tested the oncolytic activity of the KNTc-gD:GDNFΔ38 virus in a subcutaneous MCF7 flank tumor model in athymic nude mice. At 22 days post cell implantation, established tumors (average volume 70 mm^3^) were injected once with 1 × 10^8^ pfu of virus and tumor volumes were recorded every 2–3 days for 85 days. While phosphate-buffered saline (PBS)-treated tumor sizes increased steadily over this time to a final volume of ~2000 mm^3^, virus-treated tumors regressed rapidly and the animals were tumor-free at the end of the observation period (Figure 6C).

## 3. Discussion

HSV-derived oncolytic vectors have been tested in clinical trials for the treatment of solid tumors, including breast cancer [6]. The oHSV Imlygic, currently approved for the treatment of melanoma, was well tolerated in a clinical trial that included 14 metastatic breast cancer patients and reported evidence of tumor cell necrosis [3]. Another HSV-based vector, HF10, was tested in a clinical trial in metastatic breast cancer patients. It too was found to be safe, while demonstrating variable amounts of cancer cell death [4]. The apparent safety of HSV as an oncolytic agent via direct intratumoral injection and the resultant tumor cell killing represent an incentive for further refinement of HSV as a platform for breast cancer oncolytic therapy.

Breast cancer cases are heterogenous, characterized by distinct gene expression profiles, and this heterogeneity defines the course of treatment and patient prognosis. Dependent upon the tumor type, standard of care for breast cancer typically includes one, or a combination, of surgery, chemotherapy, radiation therapy, hormonal therapy, and more recently, targeted-antibody or small-molecule therapy. For example, ER^+^ cancers are commonly treated with Selective Estrogen Receptor Modulators (SERMs) or Selective Estrogen Receptor Downregulators (SERDs) that antagonize ER function and are anti-proliferative and apoptotic. However, in over 50% of cases, patients acquire resistance via mechanisms, such as loss of ER expression or alterations in growth factor signaling pathways [38,39]. The cell heterogeneity observed within individual tumors prior to treatment [40,41], and the drift that can occur throughout the course of standard treatment protocols, suggest that complementary or alternative therapies are needed and that a combination of targeted therapies may be more effective than just a single vector or drug. HSV-based, retargeted oncolytic vectors that specifically recognize co-expressed tumor-associated cell-surface proteins provide a unique therapeutic approach to cancer care that can be designed to complement current treatments. HSV-infected cells lyse rapidly, eliminating opportunities to downregulate the targeted receptor.

The receptor tyrosine kinase RET (“REarranged during Transfection”) and its coreceptors of the GDNF family, including GFRα1, are frequently upregulated in ER^+^ breast cancer cases [39]. RET and GFRα1 are implicated in promoting breast cancer-cell survival, proliferation and migration and in vitro data support the involvement of RET signaling pathways in development of drug resistance [27,39,42,43]. Elevated GFRα1 expression in breast cancer has also been linked to lymph node metastasis and poor prognosis [44]. RET and its coreceptors therefore represent viable therapeutic targets for use in conjunction with current breast cancer therapies, and GFRα1 has recently been assessed as a target for antibody-drug conjugates [25,45]. In this study, we created an HSV vector that is specifically retargeted to GFRα1, extending the current repertoire of PSMA-, EpCAM-, EGFR- and HER2-retargeted HSVs [15,16,17,18].

Cell lines derived from breast cancer patients can be classified into subtypes based on receptor expression, similar to those identified in patients [46,47]. Of the cell lines we analyzed, MCF7 and HCC1500 cells represent luminal A-type cells (ER^+^ and HER2^-^), while MDA-MB-453 are HER2 overexpressing (ER^-^ and HER2^+^) and MDA-MB-231 are basal-like (ER^-^ and HER2^-^). We confirmed that only the ER^+^ MCF7 and HCC1500 cell lines express GFRα1, raising the expectation that our GFRα1-specific oHSV will seek out a distinct subtype of breast cancer cells than the previously developed HER2-retargeted oHSVs. Indeed, we demonstrated that the retargeted virus efficiently infected and killed GFRα1-positive, but not GFRα1-negative, breast cancer cells in vitro, and knockdown of GFRα1 protein expression significantly reduced retargeted virus infection. Furthermore, we showed that this virus can mediate long-term GFRα1^+^ tumor regression upon intratumoral injection.

Tumor receptor-specific oncolytic viruses could be particularly beneficial if systemic administration results in their preferential localization to both the primary tumor and dispersed metastases. In breast cancer patients, elevated GFRα1 expression has been linked to tumor lymph-node metastases [27,44,45] and ultimately, the goal of oHSV targeting to GFRα1-expressing cells is therefore to enable safe and effective system-wide treatment. Thus far, we have not observed preferential homing to MCF7 subcutaneous tumors following intravenous injection of the retargeted virus. Multiple factors may affect vector delivery via this route. In particular, the genome copy to pfu ratio of our virus stock (Table 1) suggests that a substantial fraction of the particles in this preparation is unable to enter and/or spread within receptor-bearing cells. This may be due to reduced incorporation of the modified gD protein into the viral envelope. As discussed in Tuzmen et al. [35], it is unclear how much gD is required for effective infection, particularly when delivered intravenously. As a potential remedy, our current work seeks to increase retargeted gD incorporation into virions by systematic analysis of rational modifications to its design. If successful, our HSV-based approach to targeted breast cancer therapy will not only single out receptor-expressing cells for oncolysis, but also provide abundant payload capacity to co-deliver transgenes that can augment therapy, such as immune-modulatory genes that counteract the immunosuppressive TME [48].

## 4. Materials and Methods

### 4.1. Cell Lines

MCF7 cells were obtained from Adrian Lee (University of Pittsburgh). MDA-MB-453, HCC1500 and MDA-MB-231 cells were obtained from ATCC (Manassus, VA, USA). HCC1500 cells were cultured in RPMI media (ATCC) supplemented with 10% fetal bovine serum (FBS). MCF7 and MDA-MB-453 cells were cultured in Dulbecco’s modified Eagle’s medium (DMEM; Corning, Durham, NC) supplemented with 10% FBS. The U2OS-ICP4 cell line [49] was cultured in DMEM supplemented with 10% FBS and 2 μg/mL puromycin (ICP4 selection). Baby hamster kidney J1.1-2 cells were provided by Gabriella Campadelli-Fiume (University of Bologna) and murine melanoma B78H1 cells were provided by Gary Cohen (University of Pennsylvania); both cell lines were cultured in DMEM supplemented with 5% FBS. Nectin-1 transduced J-C cells and B78-C cells were cultured in DMEM supplemented with 5% FBS and antibiotic selection as previously described [50]. J-GFRα1 and U2OS-ICP4-GFRα1 stable cell lines were generated by transfection of J1.1-2 cells and U2OS-ICP4 with pCMV6-Kan/neo-GFRa1 and selection with 400 μg/mL or 700 μg/mL G418, respectively. The B78-GFRα1 stable cell line was generated by infection of B78H1 cells with GFRα1 retrovirus and selection with blasticidin (2 μg/mL). Retrovirus production was performed as described [49]. In each case, single clones were isolated, screened by immunofluorescence with anti-GFRα1 antibody, and positive clones were expanded for this study (Figure S1).

### 4.2. Plasmids

pCMV6-Kan/neo-GFRa1 was obtained from Origene (Rockville, MD, USA) (MC203605, untagged murine GFRα1). The cDNA for murine pre-pro-GDNF was obtained from Thermo Scientific (Waltham, MA, USA) (pCR4-GDNF; MMM1013-99829087). To obtain the pCX4-GFRα1-bsr retroviral plasmid, first a pCX4-GW-bsr plasmid was constructed by insertion of a GW cassette into pCX4-bsr [51] as previously described [49]. We then subcloned the GFRα1 coding sequence from pCMV6-Kan/neo-GFRa1 into pENTR1A (Thermo Scientific, Invitrogen) and recombined the GFRα1 sequence into pCX4-GW-bsr by LR clonase II (Thermo Scientific, Invitrogen) reaction. The pENTR-gD:wt plasmid contains the wild-type (wt) gD coding sequence (strain KOS) between the *att*L1 and *att*L2 recombination sites in pENTR1A. The gD coding sequence from the previously described pgD:∆224/38C plasmid [22] was transferred to pENTR1A and the Y38C point mutation located between BstB1 and BspE1 sites was replaced with the ∆38 mutation as described [35] to generate pENTR-gD:∆224/∆38; all retargeted gD constructs were derived from this plasmid. In pENTR-gD:GDNF∆38, the N-terminal coding sequence of gD:∆224/∆38 up to gD codon 25 was replaced with the murine pre-pro-GDNF coding sequence obtained from pCR4-GDNF by PCR with primers GDNFDraIF and GDNFSpeIR (Table 2). To create the pENTR-gD:GDNF construct, a fragment containing codon 38 was amplified from pENTR-gD:wt with primers BstBIY38F and BspEIY38R (Table 2) and cloned between the BstBI and BspEI sites of pENTR-gD:GDNF∆38. All constructs were confirmed by DNA sequencing.

### 4.3. Viruses

The BAC-containing gD-null viral backbone, KNTc-ΔgD:GW (Figure 1A), was derived from KNTc BAC [49] by Red-mediated replacement of the gD coding sequence with a GW cassette that had been amplified with RED-gD-delF and RED-gD-delR primers (Table 2) targeting the proximal 5′ and 3′ gD untranslated sequences, essentially as described [35]. Wt and retargeted gD genes were then introduced by LR Clonase II-mediated recombination of the GW cassette with different pENTR-based gD plasmids [52]. Recombinants were confirmed by field inversion gel electrophoresis (FIGE) of restriction digests followed by PCR and DNA sequencing across the gD cassettes. Infectious viruses were produced by transfection of U2OS-ICP4-GFRα1 cells and biological titers were determined by standard plaque assays (Table 1).

### 4.4. Genome Copy Titers

Viral genome copy (gc) titers were determined essentially as described [49] (Table 1). Briefly, viral DNA was collected using the DNeasy Blood and Tissue Kit (Qiagen, Germantown, MD, USA) and gc numbers were determined by qPCR for the gD gene relative to a standard curve. The portion of the gD gene amplified in this assay corresponded to an unchanged region roughly 100 codons downstream of codon 38.

### 4.5. Flow Cytometry

3 × 10^5^ cells were seeded in a 6-well dish and infected with virus at MOI 3. At 24 hpi, cells were washed with PBS and dispersed with enzyme-free cell dissociation reagent (Thermo Fisher Scientific, Gibco). Cells were fixed in 2% PFA, washed 3 times with FACS buffer (1% BSA in PBS), filtered through a 45-micron filter and stained with primary antibody against gB (Virusys, Taneytown, MD, USA) and anti-mouse Alexa488-labeled secondary antibody (Molecular Probes, Eugene, OR, USA). Fluorescent cells were detected on a BD Accuri C6 flow cytometer (BD Biosciences, San Jose, CA, USA) and data was analyzed with FloJo v10 software (FlowJo LLC, Ashland, OR, USA). Uninfected cells were used as controls.

### 4.6. Western Blot

Whole cell lysates were collected in 1× RIPA buffer (MilliporeSigma, Burlington, MA, USA) plus protease inhibitor cocktail (MilliporeSigma, Roche) and samples were diluted in 1× Laemmli sample buffer (BioRad, Hercules, CA, USA). Viruses were diluted in 1× Laemmli sample buffer at 1 × 10^8^ gc/well. Lysates were heated for 5 min at 100 °C, proteins were separated by polyacrylamide gel electrophoresis and transferred to PVDF membrane. The membrane was blocked for 1 h in 5% nonfat dry milk in PBS + 0.05% Tween and incubated sequentially with primary antibody and horseradish peroxidase-conjugated secondary antibody (anti-mouse IgG; Abcam, Cambridge, UK). Primary antibodies: gD (DL6) (Santa Cruz, Dallas, TX, USA), gB (Virusys), GFRα1 (Thermo Fisher Scientific), β-actin (Abcam).

### 4.7. AlamarBlue Cell Viability Assay

3 × 10^3^ cells were seeded in 96-well dishes 24 h prior to infection. Cells were infected at 3 pfu/cell in 60 μL serum-free media at 37 °C for 1.5 h and overlaid with 60 μL media containing 10% FBS. Wells without cells and uninfected cells were used as controls. At 24, 48, and 72 hpi, 10 μL alamarBlue reagent (Thermo Fisher) was added to the cells and the plates were incubated for a further 3–5 h at 37 °C. Supernatants were transferred to opaque-black 96-well dishes and samples were measured for fluorescence by a Biotek (Winooski, VT, USA) plate reader (560 nm excitation/590 nm emission).

### 4.8. siRNA Transfection

2 × 10^4^ cells were seeded in a 48-well dish 24 h prior to transfection. Cells were transfected with siGenome human GFRα1 SMARTPool siRNA or siGenome non-targeting pool control siRNA (Horizon, Cambridge, UK) using DharmaFECT reagent 1 at a final siRNA concentration of 25 nM. Protein samples for Western blot analysis were collected 72 h post-transfection, and parallel wells were infected in triplicate with KNTc-gD:GDNF∆38 virus at 2 pfu/cell or KNTc-gD:wt virus at 0.2 pfu/cell in 120 μL serum-free media for 1.5 h at 37 °C and overlaid with 120 μL media containing 10% FBS. At 6 hpi, cells were fixed in 4% paraformaldehyde and cells were stained with mouse primary antibody against ICP4 (Santa Cruz) and Alexa 488-conjugated secondary antibody (Thermo Fisher Scientific); nuclei were stained with DAPI. Images were taken for each infection and stained cells were counted using ImageJ software version 1.52. ICP4-positive cells were counted and calculated as the percentage of DAPI stained total cells.

### 4.9. Animal Studies

5 × 10^6^ MCF7 cells were injected subcutaneously in the right hind flank of six female BALB/c athymic nude mice (Jackson Labs, Bar Harbor, ME, USA). When the tumors reached an average of 70 mm^3^ (d22), PBS (*n* = 3) or 1 × 10^8^ pfu of KNTc-gD:GDNF∆38 virus (*n* = 3) was injected intratumorally (single dose/animal). Tumors were measured every several days for 85 d by an animal technician who was blinded to the experimental details, and volumes calculated as (L × W^2^) × 0.52. If tumor dimension in one orientation exceeded 20 mm, or the tumor volume exceeded 2000 mm^3^, the mice were humanely euthanized. If mice were unable to ambulate, eat, or drink, lost > 10% body weight, or showed a disheveled appearance they were humanely euthanized. If the cell or virus injections induced redness and inflammation at the injection site, topical antibiotic was administered to minimize risk of topical pathogen infection. All animal studies were approved by the University of Pittsburgh Institutional Animal Care and Use Committee (IACUC protocol 19024419) in accordance with the requirements and recommendations in the NIH Guide for the Care and the Use of Laboratory Animals (Institute for Laboratory Animal Research, 1985).

### 4.10. Statistical Analysis

GraphPad Prism 8 software for MacOS was used for all statistical analyses. Averages for each experiment were calculated ± SD or SEM, as indicated in the respective figure legends. Two-way ANOVA analyses were used to determine the statistical significance of differences observed between groups.

## Figures and Tables

**Table 1:** Viral Titers.

Virus	pfu/mL ^1^	gc/mL ^2^	gc/pfu Ratio
KNTc-gD:GDNF	9 × 10^9^	1.6 × 10^12^	180
KNTc-gD:GDNFΔ38	2.3 × 10^8^	1 × 10^12^	4347
KNTc-gD:wt	1.9 × 10^9^	4.2 × 10^11^	224

^1^ Viral titers in pfu/mL on U2OS-ICP4-GFRα1 cells. ^2^ Viral titers in genome copies (gc)/mL.

**Table 2:** Primer Sequences.

Primer Name	Primer Sequence
GDNFDraIF	GTCAGATTTAAAATGGGATTCGGGCCACTTGGAG
GDNFSpeIR	GTCAGAACTAGTAGAGCCTCCACCTCCAGATCCTCCACCGCACTGCCACCTCCGCCGATACATCCACACCGTTTAGCGG
BstBIY38F	CGGGGGTTCGAAGAGTGTACCACATCCAGGCGGGCCTAC
BspEIY38R	GTTGTTTCCGGACGTCTTCGGAGGC
RED-gD-delF ^1^	CCCGATCATCAGTTATCCTTAAGGTCTCTTTTGTGTGGTGCGTTCCGGTacaagtttgtacaaaaaagctgaac
RED-gD-delR ^1^	CATCCCAACCCCGCAGACCTGACCCCCCCGCACCCATTAAGGGGGGGTATaccactttgtacaagaaagctgaac

^1^ gD homology arms are specified in uppercase letters and GW cassette primer binding regions are specified in lowercase letters.

## Supplementary Materials

The following are available online at https://www.mdpi.com/1422-0067/21/22/8815/s1.
