# Supplementary material for: Generation of an Oncolytic Herpes Simplex Viral Vector Completely Retargeted to the GDNF Receptor GFRα1 for Specific Infection of Breast Cancer Cells
Source: Int J Mol Sci. 2020 Nov 21;21(22):8815. doi: 10.3390/ijms21228815 (PMC7700293; doi:10.3390/ijms21228815)
Supplement: Supplementary file 1 [file ijms-21-08815-s001.pdf]

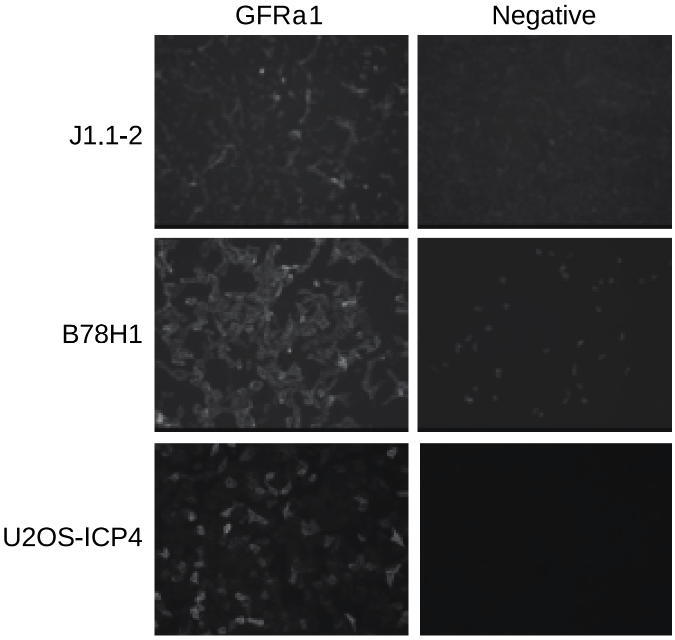

Figure S1. GFRα1 transduced cell lines. J1.1-2, B78H1 and U2OS-ICP4 cell lines were transduced with GFRα1 expression constructs, stable clones were selected by antibiotic selection and screened by immunostaining with anti-GFRα1 antibody. Representative immunostaining results for the stable clones selected for this study are shown in comparison with the GFRα1-negative parental cell lines.

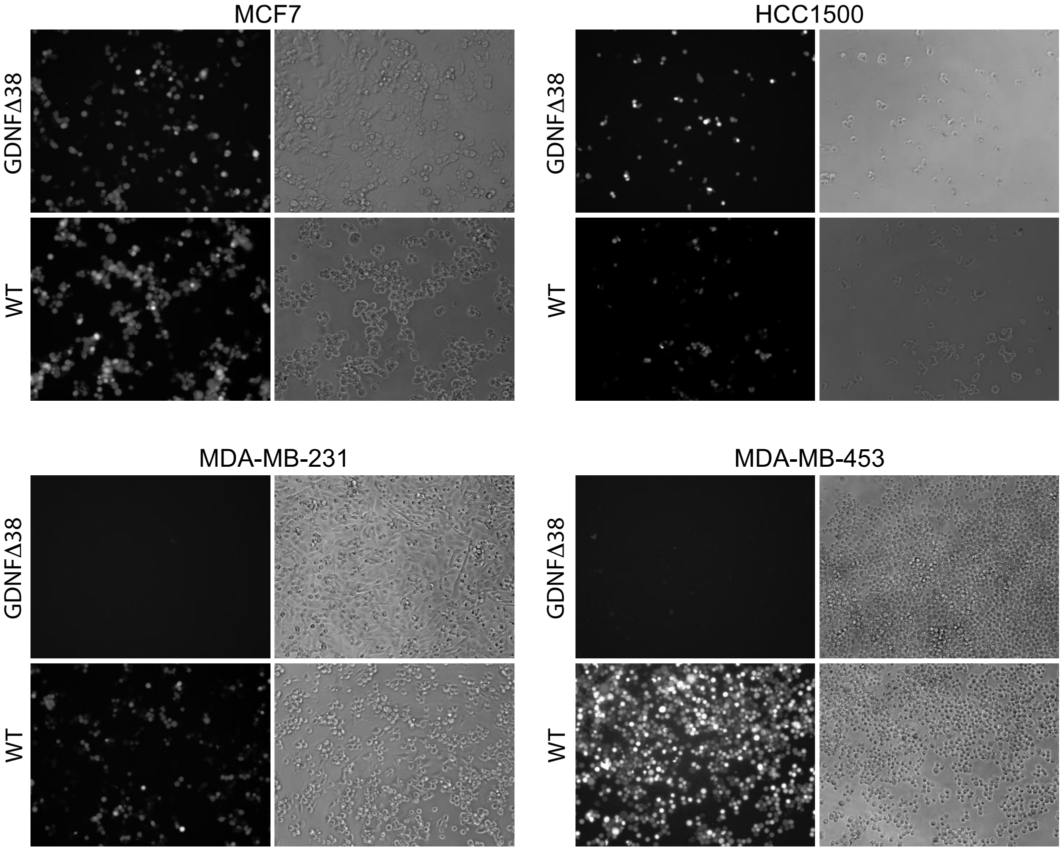

Figure S2. Virus entry into breast cancer cell lines. MCF7, HCC1500, MDA-MB-231 and MDA-MB-453 cell lines were infected with KNTc-gD:GDNFΔ38 or KNTc-gD:wt virus at 5 pfu/cell and mCherry expression was visualized at 24 hpi. Brightfield images show cell morphology and cell density at the time of imaging.
